# The Institutional Worker

**Published:** 1920-12-11

**Authors:** 


### INSTITUTIONAL WORKER

Being a Special Supplement to "The Hospital"

### OUR BUREAU OF INFORMATION.

### Rules for Correspondents

1. Every letter must be accompanied by the coupon to be cut from the back cover (inside page) of The Hospital, current issue, and must contain the name and address of the correspondent with pseudonym for publication if desired. Replies by post cannot be given save under exceptional circumstances at the Editor's discretion.

2. Letters from Approved Homes in reply to special needs published in the Bureau should state terms and full particulars, and be sent prepaid under cover to the Editor of the Bureau with name written across coupon for identification.

3. Proprietors of Homes which have not yet been entered on the List of Approved Homes, but have spare accommodation likely to suit special needs, are invited to write for an application form for registration. The fee for registration, which includes two announcements of the Home in the Bureau and other privileges, is 10s.

4. All communications to be addressed to the Editor of The Hospital, 28 Southampton Street, Strand. London, W.C. 2, and marked "Bureau of Information."

### INSTITUTION FACTS AND FIGURES.

### A Lesson from Overseas.

BY A DOMINION CORRESPONDENT.

As a worker of over twenty years' standing in a big general hospital in the Antipodes, I have thought that a few lines describing the excellent methods used in my late hospital in making the institutional workers happy and bringing a new light into their life of daily routine might be of use to our colleagues in other less understanding institutions. The hospital authorities have at all times encouraged the recreations of their servants by taking an active and genuine interest in these recreations. and have always shown that they had at heart the welfare and progress of their employees. Most of our recreations in the organisation of which we received support were pastimes in which individual skill could assert itself, and this spirit of healthy competition was fostered and encouraged by prizes presented by the hospital. Both types of mind were attracted, since equal attention was given to pursuits classed either as scientific or muscular. Some of us joined in both types, but usually a man who was good at one type devoted his whole time to it. I need not describe in detail the societies and clubs we formed, but the fact that we had a photographic society, and that much of its work comprised the really difficult microphotography, will illustrate the specialisation we were, under encouragement, able to develop. Also one very successful section was our chess club, and a variety of seasonal spare-time occupations made a world of difference in our social life together. The muscular recreations need no description, but the hospital assisted us to build our common room, in which most of our indoor games were held, and other muscular recreations we were allowed to take on the students' playing fields at certain stated times in the week. Generally speaking, I have never since met with a more harmonious industrial atmosphere than our little band represented, nor need I add that we were a very contented and, I believe, truly efficient staff. That we were content with our lot is shown by the fact that on retirement many of our members had over thirty-five years' service to their credit. Space would not allow of my detailing all the small but, to us, important arrangements we had to make. but the lesson was to me always clear that sympathetic understanding will make the most diversely constituted team pull together like one man—and in the hospital world it is the only way.

### Sphere-Gap Voltmeter.

Some importance is now attached to the measurement of voltage applied to an *x*-ray tube. The penetrative quality of the ray depends upon the E.M.F. at the poles of the tube. Ordinarily the degree of penetration is measured by an instrument placed under the fluorescent screen which shows the relations of shadow density between a thin piece of metal offering slight opacity to the rays and another piece of metal the gauge of which is graduated. (Silver and aluminium are generally used for this purpose.) To test a tube with this instrument takes up a good deal of time and requires considerable judgment. A more convenient method largely employed is to judge the E.M.F. of the discharge by the length of the air-gap it will bridge. This is not a very accurate method, for a larger or shorter air-gap between two points may be bridged by a similar E.M.F., in accordance with the frequency of discharge and atmospheric conditions. The Bauer qualimeter, a uni-polar instrument on the principle of the gold-leaf electroscope, the readings of which are really records of varying E.M.F. at the poles of the tube, is a convenient and useful instrument and, as a rule, is dependable. The voltmeter is the ideal instrument, but, unfortunately, the manufacture of one suitable for the measurement of the high E.M.F. required in *x*-ray work presents many difficulties. Messrs. Watson & Sons (Electro-Medical), Ltd., have now placed on the market an instrument known as the sphere-gap voltmeter. Subject to certain precautions, it is said to be a reliable instrument capable of measuring voltages from, say, 10,000 to 50,000, with only about 2 per cent. of error. The construction of this instrument has been based upon the observations that frequency and wave-shape have no appreciable effect when the discharge is between spheres instead of points; connections for atmospheric conditions are readily made, and a table for this purpose is provided. It has been found desirable to limit the length of the gap to the diameter of one of the spheres. The prices of this instrument range from £10 10s. to £20, in accordance with the type.

### Maternity Home Fees.

Bolton Corporation Health Committee has fixed charges in connection with the Haslam Maternity Home as follows: For private room with single bed, £5 5s. per week; patient to pay fees of private doctor if one is desired. For double-bedded room, joining with another person, £4 4s. per week; patient to pay fees of private doctor if one is desired.

For general cases the fees will be: If husband and wife are insured under the National Insurance Act, £2 2s. per week; if only either husband or wife is insured, £1 1s. per week.

These fees may be reduced at the discretion of the medical officer of health under special circumstances. All other cases not coming under the above classes will be dealt with at the discretion of the medical officer of health.

### Question for December.

### THE WORKING OF THE NATIONAL HEALTH INSURANCE ACTS.

**What benefits, if any, have hospitals derived from the National Health Insurance Acts? Have they been helpful in the management of the Almoners' or Enquiry Officers' Department, and if so how?**

**(A minimum payment of 10s. 6d. will be made for each published answer.)**

### RULES.

**The following rules must be observed:—**

**1. Contributions must be written on one side of the paper. Conciseness and terseness are desirable features. The MS. must bear the name and address of the sender and be accompanied by coupon to be cut from the back cover (inside page) of the current issue of The Hospital. A pseudonym must be chosen if the name is not to be published.**

**2. Contributions must be addressed to the Editor of The Hospital Institutional Worker Supplement, 28 & 29 Southampton Street, Strand, London, W.C. 2, should reach him before the end of the current month, and be marked in the left-hand corner "Facts and Figures."**

### QUESTIONS INVITED.

In connection with our Question Box, we offer every month five shillings for the best question sent in for consideration. The questions may be on any institutional subject and concern **any department, from the secretary's to the porter's, or the matron's to the domestic staff; the one essential is that they must be practical and deal with points that have a definite relation to hospital or institutional administration, upkeep, finance, or management. Points relating to artificers' work, housekeeping, the laundry, out-patients, and other practical matters will be welcome. It is hoped that thus, when difficult or doubtful points arise in the course of their work, institutional workers will be encouraged to put them in the form of a question and send them to the Editor, The Hospital Bureau of Information, 28 Southampton Street, Strand, W.C. 2, marked "Question Box."**

**The best questions will be published in due course and our readers will be given the opportunity of answering them. Thus all institutional workers may be able to co-operate with the view of helping the work and smoothing the difficulties of each other. In short, we want the Question Box of the *Institutional Worker* to be freely used by every worker habitually.**

### ENQUIRIES AND ANSWERS.

### *THE SICK AND IN NEED.*

### Home for Incurables for the Middle Classes.

We suggest that you write to the Secretary of the British Home and Hospital for Incurables at 72 Cheapside, E.C. 2, for a form of application with a view to entering the home. This home is for the middle classes. Admission is upon election of subscribers, and prospective candidates must not be under thirty-five years of age. Elections take place in May and November. The Secretary, no doubt, will give you all other necessary information when you write to him.—In Need.

### Home for Child of a Clergyman's Widow.

The Infant Orphan Asylum, Wanstead, E., will probably be able to help you by admitting your child of five years to their home. Admission is by election of subscribers. Write to the Secretary of the Asylum at 63 Ludgate Hill, London, E.C., who will give you all necessary information.—Clergyman's Widow.

### Convalescent Home for Boy.

We think the Children's Sick and Convalescent Home, London Road, Luton, would be fairly near to you. Boys are accepted for admission between the ages of three and eight years. Admission is by subscriber's letter and payment of 2s. 6d. per week. Children are allowed to stay as long as two months, and even this period may be prolonged. The Hon. Secretary will possibly help you to obtain a letter. Write to him and inquire.—Mother.

### Hospitals for Paying Patients.

We give you the addresses of a few hospitals for paying patients in London: The Empire Nursing Home, Vincent Square, Westminster, S.W.; Home Hospitals Association, Fitzroy Square, W.; and the Florence Nightingale Hospital for Gentlewomen, 19 Lisson Grove, N.W. 1. Many general hospitals in London have beds set aside for the use of private patients, such as the Great Northern Central Hospital, Holloway Road, N.; Hampstead General Hospital, Haverstock Hill, N.W.; Guy's Hospital, S.E.; the St. Thomas's Home (in connection with St. Thomas's Hospital), Albert Embankment, S.E. 1; and the South London Hospital for Women, South Side, Clapham Common.—Mrs. C.

### Jewish Hospital.

There is the London Jewish Hospital at 51a Stepney Green, E. 1. Write to the Secretary for details of admission. The London Hospital, Whitechapel Road, E., has special wards for Jewish patients.—Hebrew

### *EMPLOYMENT AND TRAINING.*

### Cottage Nursing.

Write to the Secretary of the Cottage Benefit Nursing Association, Denison House, 296 Vauxhall Bridge Road, S.W. 1, for particulars of training of cottage nurses on the Holt-Ockley system. We regret we cannot give postal replies; a coupon should be enclosed with each inquiry.—H. C.

### Prison Nursing.

Apply to the Home Office, Whitehall, S.W., for particulars of prison nursing. You would not be acceptable for work in a private institution without a general-hospital training, but with your certificate for fever nursing you could continue your work in fever hospitals.—E. M. J.

### Practical Experience Required in Midwifery.

Medical pupils can obtain practical experience at Queen Charlotte's Lying-in Hospital, Marylebone Road, N.W., for periods ranging from four weeks to twelve weeks, and the fees vary from £8 8s. upwards. Write for terms, rules, etc., to the Secretary at the hospital.—Medico.

### Training in the Administration of Anæsthetics.

Write and inquire if you can obtain training in the administration of anæsthetics at the Royal Victoria Hospital, Newcastle-on-Tyne, or, if you do not object to London, write to the Missionary School of Medicine, 82 Wimpole Street, W. 1, and the Mildmay Mission Hospital, Bethnal Green, E. Enclose stamped addressed envelope for reply.—Babs.

### Tropical Medicine.

The fees for a three months' session at the London School of Tropical Medicine (University of London), Royal Albert Docks, E. 16, is £16 16s. Write for further particulars to the Secretary, P. J. Michelli, C.M.G. The fees for a full course at the Liverpool Incorporated School of Tropical Medicine, H 24-25 Exchange Buildings, are £13 13s.—M. D.

### Maternity and Child Welfare Work.

The Maternity Charity and District Nurses' Home, Howard Road, Plaistow, London, E., accept candidates for training in midwifery and child-welfare work. The Secretary will give you full particulars regarding terms of training upon application. Enclose a stamped addressed envelope for reply.—Dora.

### *MISCELLANEOUS.*

### Handbook for Tuberculosis Nurse.

Obtain the new "Handbook on Tuberculosis," by G. Norman Meachen, M.D., which you will doubtless find most useful to you. It is published by The Scientific Press, Ltd., 28 and 29 Southampton Street, Strand, W.C. 2. The price is 1s. 4½d., post free.—T. A. S.

